# Antimicrobial Susceptibility Patterns and Outcomes of Neonatal Early-Onset Sepsis over a Decade: Implications for Empirical Therapy in a Tertiary NICU

**DOI:** 10.3390/jcm15062103

**Published:** 2026-03-10

**Authors:** Katarzyna Muszyńska-Radska, Joanna Kwiecińska-Piróg, Iwona Sadowska-Krawczenko

**Affiliations:** 1 Department of Neonatology, Faculty of Medicine, Ludwik Rydgier Collegium Medicum in Bydgoszcz, Nicolaus Copernicus University in Toruń, Ujejskiego 75, 85-168 Bydgoszcz, Poland; iwonasadowska@cm.umk.pl; 2 Department of Microbiology, Faculty of Pharmacy, Ludwik Rydgier Collegium Medicum in Bydgoszcz, Nicolaus Copernicus University in Toruń, M. Skłodowskiej-Curie 9, 85-094 Bydgoszcz, Poland; j.kwiecinska@cm.umk.pl

**Keywords:** neonatal sepsis, early-onset sepsis, empirical therapy, antimicrobial susceptibility, antimicrobial resistance

## Abstract

**Background:** The goal of this study was to characterize the microbial etiology, antimicrobial susceptibility, and temporal resistance trends of early-onset neonatal sepsis (EOS) pathogens in a tertiary neonatal intensive care unit over 10 years (2014–2023), assessing empirical therapy adequacy and mortality associations. **Methods:** Retrospective analysis was performed on the positive blood cultures of neonates with confirmed EOS, born between 1 January 2014 and 31 December 2023. Blood was aseptically collected into PEDS Plus/BC bottles, incubated using the BACTEC system, with pathogen identification by biochemical assays or MALDI-TOF MS. Susceptibility testing followed EUCAST disk-diffusion standards, with additional resistance assays. **Results:** Among 6631 NICU admissions, 39 neonates met EOS criteria (31 preterm, 8 term). In preterm infants, Gram-negative *Enterobacterales*—mainly *E. coli* (*n* = 20)—predominated, while GBS was most common in term infants. All GBS isolates (*n* = 7) were susceptible to benzylpenicillin and vancomycin. Although 90% of *E. coli* were ampicillin-resistant, 90–95% remained susceptible to third-generation cephalosporins, piperacillin–tazobactam, and aminoglycosides. Two *E. coli* isolates produced ESBL but remained susceptible to aminoglycosides and carbapenems. Mortality was higher in *E. coli* EOS (50%) than in GBS (0%) or other pathogens (25%), with borderline significance (*p* = 0.0547; adjusted RR 1.55, 95% CI 0.54–4.41). Ampicillin resistance was not associated with increased mortality. No annual resistance trends were observed. **Conclusions:** In this 10-year NICU cohort, the etiology of EOS differed markedly between preterm and term neonates. Recommended empirical ampicillin–aminoglycoside therapy demonstrated in vitro efficacy against most neonatal bloodstream isolates pending pathogen identification. However, the widespread ampicillin resistance, particularly among *E. coli* strains, supports consideration of cephalosporin–aminoglycoside combinations or meropenem monotherapy when rapid beta-lactam bactericidal activity is clinically essential. Mortality was higher in *E. coli* EOS, though not statistically significant, and unrelated to ampicillin resistance.

## 1. Introduction

Early-onset sepsis (EOS) remains one of the leading causes of neonatal morbidity and mortality [1]. Among term newborns, its incidence is estimated at approximately 1–2 cases per 1000 live births, with a mortality rate of around 3% [2,3]. In contrast, preterm infants experience a tenfold higher incidence, with mortality rates reaching up to 50% [2,4].

Diagnosing early-onset neonatal sepsis (EOS) is often difficult due to the nonspecific nature of initial clinical symptoms and the limited sensitivity and specificity of current laboratory tests. Nonetheless, timely initiation of antibiotic therapy is crucial for improving neonatal outcomes [5]. Empirical treatment is commonly started before blood culture results—the gold standard for sepsis diagnosis—are available [6]. Current recommendations from PTN (Polish Neonatology Society), AAP (American Academy of Pediatrics), and NICE (National Institute for Health and Care Excellence) advocate for the use of ampicillin or benzylpenicillin in combination with an aminoglycoside, typically gentamicin, as first-line empirical therapy for EOS [3,7,8]. Once a positive blood culture is obtained, treatment should be adjusted according to the antibiogram. However, in a significant proportion of cases—despite clinical signs of sepsis—blood cultures remain negative, and empirical antibiotic therapy is prolonged unnecessarily, even when it should be discontinued [9,10]. This practice contributes to poorer outcomes, particularly among preterm infants, and may have long-term consequences [10]. It is also associated with serious complications such as necrotizing enterocolitis and increased mortality [11]. Prolonged antibiotic exposure is a well-recognized risk factor for the development of late-onset sepsis. Numerous studies have shown that antibiotic therapy adversely affects the composition of the gut microbiota, potentially leading to long-term health consequences, including increased susceptibility to disease later in life [10,11,12].

In recent years, there has been a marked increase in bacterial resistance, particularly among Gram-negative microorganisms such as *Escherichia coli* (*E. coli*) and *Klebsiella pneumoniae* (*Kl.pneumoniae*), which are increasingly acquiring resistance mechanisms associated with the production of extended-spectrum β-lactamases (ESBLs) and carbapenemases (CRE, carbapenem-resistant *Enterobacterales*). This issue, previously linked primarily to adult intensive care units, is now gaining particular relevance in the neonatal population, where infections caused by multidrug-resistant strains are becoming more frequent [13]. In the context of escalating global antibiotic resistance, analyzing the antimicrobial susceptibility of strains isolated from neonates with sepsis is essential for developing effective therapeutic strategies and updating empirical treatment protocols. Monitoring local resistance trends and integrating microbiological findings into clinical practice are critical components of rational antibiotic therapy, enabling the reduction in last-line antibiotic use and improving outcomes for the youngest patients [14].

This study aims to evaluate the etiology and antimicrobial susceptibility profile of bacterial strains isolated in cases of early-onset neonatal sepsis at our center, focusing on antibiotics currently used or potentially applicable in empirical therapy. We also assessed resistance trends over the study period and their correlation with clinical outcomes and mortality. Additionally, we sought to determine whether current treatment guidelines are effective against the strains isolated in our facility.

## 2. Materials and Methods

### 2.1. Study Design

The study involved a retrospective analysis of positive blood cultures and the antimicrobial susceptibility of isolated microorganisms collected from neonates with confirmed early-onset sepsis, born between 1 January 2014 and 31 December 2023, in the Neonatal Intensive Care Unit of University Hospital No. 2 in Bydgoszcz, Poland, tertiary referral center, with 6631 admissions. Sample size determined by all eligible EOS cases over 10 years (*n* = 39 confirmed), which is typical for single-center neonatal studies.

The antibiotics analyzed are those used in empirical therapy in early onset sepsis or those that may serve as potential alternatives to standard empirical treatment.

### 2.2. Definition

Early-onset sepsis (EOS) is defined as an infection that develops within the first 72 h of life.

An episode of confirmed bloodstream infection was defined as the isolation of a single potential pathogen from an infant’s blood sample, provided that four specific criteria were met.

The first criterion required the presence of clinical signs of infection, such as respiratory distress, apnea, tachycardia or bradycardia, systemic hypotension or hypoperfusion, hypothermia or fever, seizures, hypotonia, irritability or lethargy, feeding intolerance or intestinal obstruction, neonatal jaundice, or hypoglycemia.

The second criterion involved at least one abnormal hematologic parameter, including an altered white blood cell count, an elevated immature-to-total neutrophil ratio, increased C-reactive protein concentration, or abnormal procalcitonin levels.

The third criterion was the administration of appropriate antibiotic therapy for a minimum of five days after symptom recognition and blood culture collection—particularly in cases of *Coagulase-negative Staphylococcus* (CoNS) bacteremia.

The fourth criterion required a positive blood culture obtained within the first 72 h after birth.

Samples considered to be at risk of contamination were removed from the analysis. Contamination was defined by the isolation of organisms commonly viewed as non-pathogenic contaminants, such as *Bacillus* spp., *Micrococcus* spp., or *Corynebacterium* spp.; the presence of mixed CoNS flora; or the isolation of CoNS without accompanying clinical signs of infection, as assessed by the attending neonatologist.

Infants who failed to meet all four inclusion criteria, along with those whose samples were deemed likely to be contaminated, were excluded from the analysis (*n* = 13/52, 25%).

Mortality was defined as all-cause in-hospital mortality, referring to deaths occurring at any point during the neonatal intensive care unit (NICU) stay following the diagnosis of a confirmed EOS episode.

### 2.3. Methodology of Sample Collection

A neonatologist aseptically collected approximately one milliliter of blood and placed the sample into a PEDS Plus/F culture vial (Becton Dickinson and Company, Franklin Lakes, NJ, USA). Prior to collection, the skin was disinfected with a 0.5% chlorhexidine solution. Blood for culture was collected before the initiation of antibiotic therapy.

### 2.4. Microbiological Techniques

The sample was delivered to the microbiology laboratory within two hours, where it underwent a seven-day incubation in the BACTEC system (Becton Dickinson and Company, Franklin Lakes, NJ, USA) at 35 ± 2 °C to assess microbial growth. In cases of positive results, Gram staining and selective media cultures were performed. Identification was carried out using standardized biochemical tests and the MALDI-TOF MS (Matrix-Assisted Laser Desorption/Ionization–Time of Flight–Mass Spectrometry) method (Bruker Daltonik GmbH, Bremen, Germany), which employs mass spectrometry for microbial detection.

### 2.5. Antimicrobial Susceptibility Testing

Antimicrobial susceptibility testing was performed for all isolates using the standard disk-diffusion method, and both the susceptibility testing and the interpretation of results were carried out in accordance with the EUCAST guidelines applicable in the year in which each isolate was obtained [15]. Depending on the cultured microorganisms, Mueller–Hinton agar or Mueller–Hinton agar supplemented with 5% horse blood and B-NAD (in 2014–2020, bioMérieux SA, Marcy-l’Etoile, France, in 2021–2023, BioMaxima S.A., Lublin, Poland) was used for the disk-diffusion antibiogram. All discs were obtained from Oxoid, Thermo Scientific, Basingstoke, United Kingdom.

For isolates suspected of producing specific resistance mechanisms, appropriate phenotypic tests were performed using Oxoid antibiotic discs and dedicated culture media (in 2014–2020, bioMérieux SA, Marcy-l’Etoile, France, in 2021–2023, BioMaxima S.A., Lublin, Poland. In the case of Enterobacterales, the production of extended-spectrum beta-lactamase (ESBL) was determined using the double-disk synergy test (DDST) (ceftazidime, cefotaxime, cefepime, amoxicillin/clavulanic acid; Oxoid, Thermo Scientific, Basingstoke, United Kingdom. Due to the susceptibility of all Gram-negative strains to carbapenems, there was no carbapenems testing performed. All strains can be defined as carbapenems-negative.

For Gram-positive bacteria such as GBS (Group B *Streptococcus*) and CoNS, resistance to macrolides, lincosamides, and streptogramins (MLS—Macrolide-Lincosamide-Streptogramin resistance) was assessed using two disks (erythromycin, clindamycin). For CoNS, resistance mechanisms to methicillin were examined using a cefoxitin disk test. For *Enterococcus* spp., resistance to glycopeptides (VRE—Vancomycin-Resistant *Enterococci*) was assessed using an E-tests (bioMérieux SA, Marcy-l’Etoile, France), which determines the MIC (Minimum Inhibitory Concentration) for vancomycin and teicoplanin. Additionally, for *Enterococcus* spp., the HLAR (High-Level Aminoglycoside Resistance) mechanism was examined using a disk impregnated with a high concentration of aminoglycosides (gentamicin 30 µg or streptomycin 300 µg).

Depending on the isolated pathogen, antibiotic discs with appropriate concentrations were used: ampicillin for *Enterococcus* spp., *Haemophilus* spp., and *Listeria monocytogenes* (2 µg); for *Enterobacterales*, ampicillin (10 µg), cefotaxime (5 µg), ceftriaxone (30 µg), amikacin (30 µg), and gentamicin (10 µg), except for *Enterococcus* spp., for which a 30-µg gentamicin disc was used. Additionally, discs containing meropenem (10 µg), piperacillin/tazobactam (30/6 µg), and vancomycin (5 µg) were applied for *Enterococcus* spp. and GBS (Oxoid). For CoNS, the minimum inhibitory concentration (MIC) of vancomycin was determined (bioMérieux).

Isolates classified in microbiological reports as susceptible (S) or susceptible, increased exposure (I) (dose depend) were considered susceptible to a given antibiotic. These antibiotics were regarded as effective against the respective microorganisms. Isolates classified as resistant (R), those with known intrinsic resistance to the antibiotic, and isolates for which there is insufficient evidence that the microorganism is an appropriate target for treatment with the agent (IE), as well as agents not recommended for therapy and for which susceptibility testing is not advised (-), were considered resistant. Susceptible isolates were considered treatable with effective therapy, whereas resistant isolates were considered not amenable to effective treatment.

### 2.6. Treatment Protocol

The early-onset sepsis treatment protocol was based on ampicillin combined with an aminoglycoside. Third-generation cephalosporins were recommended for high-risk Gram-negative bacteria caused infections, particularly if aminoglycosides were contraindicated, such as in cases of renal dysfunction. Carbapenems were reserved for infections caused by multidrug-resistant Gram-negative organisms, including ESBL-producing strains. Once culture results became available, antimicrobial therapy was adjusted according to the susceptibility profile of the isolated pathogen. The duration of treatment ranged from 7 to 14 days, depending on the causative agent, type of infection, and the newborn’s clinical condition.

### 2.7. Statistical Analysis

To compare the prevalence of pathogens between term and preterm infants, the chi-square test without Yates’ correction and Fisher’s exact test were employed. To adjust for multiple comparisons, the Bonferroni correction was applied, with statistical significance set at *p* < 0.05. Comparative analysis of antimicrobial resistance among strains causing early-onset sepsis—categorized into three microbial groups—was conducted using a two-sided Fisher’s exact test on pooled data across the entire study period. Bonferroni correction was used to account for multiple testing, with non-informative results reported with a corrected *p*-value of 1.00. For all statistical comparisons of antimicrobial efficacy, isolates categorized as susceptible (S) or susceptible, increased exposure (I) were considered susceptible, while those classified as resistant (R), exhibiting natural resistance (NR), or designated as IE or (–) were grouped together as non-susceptible.

Temporal trends in antibiotic resistance across different years were assessed using Kendall’s tau coefficient (two-sided). The test was performed only when at least three years of data were available for a given combination and temporal variability was observed; otherwise, the test was omitted. Statistical significance was defined as *p* < 0.05. Mortality analysis (defined as all-cause in-hospital mortality) was performed using the Fisher–Freeman–Halton test and Fisher’s exact test. Subsequently, the adjusted relative risk (RR) of death was estimated using Poisson regression with robust standard errors, accounting for gestational age. Additionally, the association between ampicillin resistance in *E. coli* strains and mortality was evaluated using Fisher’s exact test, and the adjusted RR of death was estimated via Poisson regression. Selection bias was minimized by including all positive blood cultures obtained within the first 72 h (*n* = 52) and by applying transparent, predefined exclusion criteria (*n* = 13), ensuring methodological consistency throughout the analysis. Statistic analyses were performed using Python (version 3.12.2) with the SciPy (version 1.15.2) and statmodels (version 0.14.2) packages.

## 3. Results

During the study period, 52 of 6631 neonates admitted to the NICU had positive blood cultures obtained within ≤72 h of life. Thirteen cases (25%) were excluded due to not meeting EOS criteria or suspected contamination. The final analysis included 39 confirmed EOS episodes, with 31 occurring in preterm and 8 in term infants. Thirteen newborns died, while the remaining infants demonstrated full clinical and laboratory recovery from the early infection.

### 3.1. Pathogens Involved in Early-Onset Sepsis

Our analysis revealed statistically significant differences in the etiology of pathogens responsible for early-onset sepsis between term and preterm neonates. In preterm infants, Gram-negative bacteria—particularly strains from the *Enterobacterales*—were predominant, whereas Gram-positive bacteria were more common among term neonates. GBS was the leading pathogen in the term group, with a statistically significant difference in its prevalence compared to preterm infants. Although *E. coli* was the most frequent pathogen among preterm neonates, no statistically significant difference in its occurrence was observed between the two groups. Table 1. shows distribution of pathogens in early-onset sepsis among preterm and term infants.

### 3.2. Susceptibility Profiles and Resistance Patterns

An analysis of antimicrobial susceptibility and resistance among pathogens causing early-onset sepsis was performed, focused on antibiotics currently used in treatment, those recommended for empirical therapy, or those with potential applicability in clinical management. Specifically, the antibiotics assessed included ampicillin, gentamicin, amikacin, cefotaxime, ceftriaxone, meropenem, piperacillin/tazobactam, and vancomycin.

All isolated GBS (100%) strains demonstrated susceptibility to benzylpenicillin. According to EUCAST recommendations, the susceptibility of GBS to ampicillin, cefotaxime, ceftriaxone, and meropenem is inferred from benzylpenicillin susceptibility. Aminoglycosides are considered unsuitable agents for the treatment of infections caused by GBS, and susceptibility testing is not recommended.

The majority of *E. coli* isolates (90%) were resistant to ampicillin. Other Gram-negative bacteria, particularly rods from the *Enterobacterales* order, also exhibited resistance to ampicillin or were naturally resistant to it. A high percentage of *E. coli* isolates (90%) were susceptible to other β-lactam antibiotics, including cefotaxime, ceftriaxone, and piperacillin/tazobactam. Additionally, all other *Enterobacterales* rods demonstrated 100% susceptibility to these antibiotics. All strains of Gram-negative bacteria were susceptible to meropenem and other carbapenem antibiotics. All Gram-negative rods, including *E. coli*, demonstrated a high rate of susceptibility to aminoglycosides, with *E. coli* showing 95% susceptibility and all other strains exhibiting 100% susceptibility to both amikacin and gentamicin. Two (10.0%) *E. coli* isolates were ESBL producers (extended-spectrum beta-lactamase) and exhibited resistance to beta-lactam antibiotics, including ampicillin, piperacillin/tazobactam, and third-generation cephalosporins. They remained susceptible to aminoglycosides and carbapenems. Table 2 presents the antibiotic susceptibility profiles of all pathogens identified in cases of early-onset neonatal sepsis.

An analysis of antibiotic resistance and resistance trends among pathogens causing early-onset sepsis was conducted for the period 2014–2023. The study focused on *E. coli* and GBS isolates, while other less frequent pathogens were grouped under “Other” due to low individual counts. *E. coli* demonstrated a statistically significant higher rate of resistance to ampicillin compared to GBS. No statistically significant differences were observed for the remaining antibiotics. Year-to-year analysis did not reveal any significant monotonic trends in resistance for the antibiotics assessed across the studied pathogens. Table 3 shows antibiotic resistance by year and pathogen.

### 3.3. Pathogen-Specific Mortality Patterns

In the overall group, mortality differences were borderline significant (*p* = 0.0547): *E. coli n* = 10/20 (50.0%), GBS *n* = 0/7 (0%), Other *n* = 3/12 (25.0%) (Table 4). Among term infants, no statistically significant differences were observed (*p* = 0.3750): *E. coli n* = 1/2 (50.0%), GBS *n* = 0/5 (0%), Other *n* = 0/1 (0%). Similarly, in the preterm group, differences did not reach significance (*p* = 0.4062): *E. coli n* = 9/18 (50.0%), GBS *n* = 0/2 (0%), Other *n* = 3/11 (27.3%). The adjusted relative risk (RR) for *E. coli* versus other pathogens in the entire group was 1.55 (95% CI: 0.54–4.41); RR comparisons involving GBS were not reported due to the absence of deaths in that category.

In the analysis restricted to *E. coli* cases, ampicillin resistance showed no association with mortality (Table 5). Fisher’s exact test in the overall cohort yielded *p* = 1.0000, and the adjusted Poisson regression model indicated a relative risk of approximately 1.01, with no statistical significance (*p* = 0.99). Findings were consistent across term and preterm subgroups (*p* = 1.0000 and *p* = 0.978, respectively), aligning with the tabulated data.

### 3.4. Susceptibility of Isolates to the Combined Action of Antibiotics for the Assessment of Empirical Therapy Effectiveness

The analysis of strain susceptibility to the combined action of antibiotics, performed to assess the effectiveness of empirical therapy, involved assigning each isolate its antimicrobial susceptibility results together with information on intrinsic resistance mechanisms.

**Figure 1:**
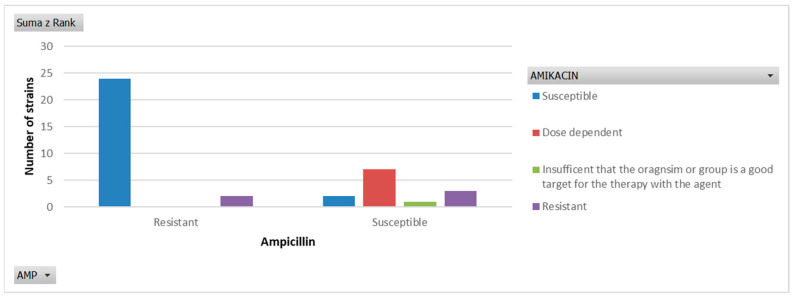
Susceptibility profile of all examined strains due to the amikacin and ampicillin.

Among all isolates obtained from neonatal bloodstream infections, only 33.3% were susceptible to ampicillin. Resistance to amikacin was identified in 5 isolates (12.8%), while in 7 isolates (17.9%) susceptibility to aminoglycosides was achievable only when high doses of the antibiotic were used. Figure 1 illustrates the susceptibility of the isolates to the synergistic activity of ampicillin and an aminoglycoside.

The highest susceptibility rate was observed for meropenem, with 94.9% of isolates remaining sensitive to this agent. Notably, isolates susceptible to meropenem were also susceptible to aminoglycosides.

**Figure 2:**
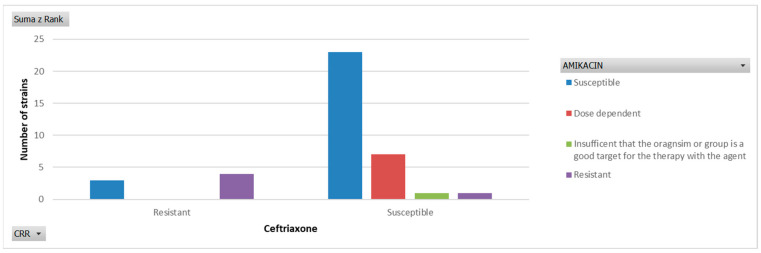
Susceptibility profile of all examined strains due to the amikacin and ceftriaxone.

The analysis showed that 82.1% of all isolates were susceptible to ceftriaxone, and as many as 89.7% were susceptible to ceftriaxone and/or amikacin. Resistance to both antibiotics was found in 10.3% of isolates, specifically: *Enterococcus* spp. (1), CoNS (1), and *Listeria monocytogenes* (1). In these rare cases, vancomycin or meropenem demonstrated therapeutic effectiveness. Figure 2 illustrates the susceptibility of the isolates to the synergistic activity of cephalosporin and an aminoglycoside.

## 4. Discussion

The results from our ten-year study provide essential insights into epidemiology, antibiotic susceptibility, resistance trends, and clinical outcomes associated with early-onset neonatal sepsis. The analysis, based on a date from 2014 to 2023, reveals significant differences in pathogen profiles and antimicrobial resistance between preterm and term neonates.

Before addressing the primary objective of our study, we conducted a preliminary analysis of pathogen distribution, which provided the basis for the subsequent discussion on antimicrobial susceptibility. This analysis focused on pathogens responsible for early-onset sepsis (EOS) in preterm and term infants, revealing significant differences in etiology between these two groups. Gram-negative bacteria, particularly *Enterobacterales*—mainly *E. coli*—were predominant among preterm neonates, whereas Gram-positive pathogens were more common in term infants. These findings are consistent with those reported by Liu et al., Stoll et al., and Miselli et al. [1,16,17]. Moreover, both Stoll and Miselli emphasize the increasing incidence of *E. coli*-related EOS in preterm infants [1,17]. Stoll et al. further highlight the need for ongoing surveillance of its rising prevalence in bloodstream infections within this vulnerable population. Their work underscores the importance of implementing preventive strategies aimed at reducing the incidence of such infections through comprehensive risk factor analysis. Identifying women eligible for prophylactic interventions, as well as determining clinical scenarios in which broad-spectrum antibiotic therapy should be administered to both mothers and neonates, may play a pivotal role in mitigating the impact of *E. coli*-related neonatal sepsis [1]. Moreover, the higher incidence of *E. coli* EOS in preterm infants (<34 weeks) may be related to reduced transplacental transfer of maternal IgG, which limits neonatal immune protection. A similar pattern has been shown for GBS, where higher levels of specific IgG (>1 µg/mL) markedly reduced the risk of EOS [18].

The primary objective of our study was to analyze the antimicrobial susceptibility of strains responsible for early-onset sepsis (EOS).

All GBS (100%) isolates demonstrated susceptibility to benzylpenicillin, which allowed inference of their sensitivity to other beta-lactam antibiotics, including ampicillin, cefotaxime, ceftriaxone, and meropenem [19]. Additionally, all GBS strains (100%) were susceptible to vancomycin. Several studies, including those by Miselli et al., Mariani et al., confirm the high susceptibility rate of GBS to ampicillin, further supporting its effectiveness in the treatment of bloodstream infections caused by this pathogen [14,17].

Our study revealed a high percentage of *E. coli* strains—one of the most common etiological factors in early-onset sepsis (EOS)—that are resistant to ampicillin (90%), which is the first-line antibiotic in empirical therapy. This trend has been confirmed by multiple studies, such as Dustin D. Flannery et al., Puopolo et al. and many more [7,19].

Other Gram-negative bacteria, particularly *Enterobacterales* rods, also exhibited resistance to ampicillin or were naturally resistant to it. The high prevalence of ampicillin resistance may partly reflect the widespread use of intrapartum antibiotic prophylaxis (IAP) to prevent vertical GBS transmission. According to Stoll et al., prolonged intrapartum exposure to beta-lactams increases the risk of resistant Gram-negative pathogens emerging in neonates [1].

Most Gram-negative bacterial strains, including *E. coli*, demonstrated approximately 95% susceptibility to beta-lactam antibiotics, such as third-generation cephalosporins (cefotaxime and ceftriaxone) and piperacillin/tazobactam. Third-generation cephalosporins serve as an alternative to ampicillin in the treatment of EOS caused by Gram-negative bacteria, including *E. coli*. Both cefotaxime and ceftriaxone share similar therapeutic indications in the management of severe neonatal infections, such as sepsis and meningitis [20]. However, antibiotic selection should be guided by individual clinical contraindications. Cefotaxime is preferred in neonates due to more robust clinical documentation and a lower risk of adverse effects. In contrast, ceftriaxone may cause serious complications, including hyperbilirubinemia and interactions with calcium-containing solutions, which limit its use during the first weeks of life, particularly in premature and high-risk infants [21].

In our ten-year observational study, only two strains producing extended-spectrum beta-lactamases (ESBL) were identified. No carbapenemase-producing strains were detected during this period, and all isolates from the *Enterobacterales* demonstrated full susceptibility to carbapenems. These findings are consistent with data reported by Dustin et al., indicating that ESBL rates in neonates remain low and carbapenem-resistant *Enterobacterales* (CRE) are rare [13].

Carbapenems, particularly meropenem, are considered the treatment of choice for infections caused by ESBL-producing organisms [13]. The efficacy of this approach has been confirmed in studies by Hariss et al., which demonstrated significantly higher mortality rates in patients treated with piperacillin/tazobactam compared to those receiving meropenem [22]. EUCAST guidelines also emphasize that piperacillin/tazobactam is not recommended in cases of bloodstream infections caused by ESBL-producing strains and suggest meropenem as the preferred alternative [15]. Due to the rarity of carbapenemase-producing strains in this patient population, as confirmed by our data, there is a lack of robust studies addressing the treatment of such infections in neonates. Existing data primarily concern adult populations [13]. The use of broad-spectrum antibiotic therapy is associated with the development of invasive fungal infections and may also serve as an independent factor contributing to the emergence of resistance mechanisms such as ESBL production or carbapenem resistance [23,24].

Most *Enterobacterales* strains, including *E. coli*, in our analysis demonstrated susceptibility to aminoglycosides—both amikacin and gentamicin. The toxicity of these agents in neonates has been confirmed in numerous studies, and their use requires therapeutic drug monitoring as well as regular assessment of auditory function and renal performance [25]. Amikacin is more frequently associated with vestibular toxicity compared to gentamicin [26], whereas gentamicin tends to exhibit slightly higher nephrotoxicity than amikacin [27]. Nevertheless, amikacin should be reserved for the treatment of late-onset infections caused by hospital-acquired Gram-negative pathogens, which are typically susceptible to this agent [28]. It must be remembered that aminoglycosides exhibit bacteriostatic activity and therefore should not be used as monotherapy in bloodstream infections, but only as part of combination therapy [15].

A statistical analysis conducted as part of a ten-year monitoring of microbial resistance to selected antibiotics did not reveal any significant upward trends in resistance, even during the COVID-19 pandemic period from 2020 to 2023. During this time, hospitals around the world—including our own—implemented stricter infection prevention and control measures, such as hand hygiene, disinfection, universal mask-wearing, and restrictions on visitors [29]. These factors played a key role in limiting the spread of resistant strains and may have contributed to the stable resistance trends observed in our neonatal unit [13].

Most studies on the impact of the pandemic on infections focus on adult intensive care units, where enhanced hygiene procedures did not lead to a reduction, but rather an increase in the number of infections. This was driven by various factors such as stress, haste, fatigue, and distraction [30]. As a result, infection control and antibiotic stewardship programs collapsed, leading to the spread of multidrug-resistant strains [29]. Newborns represent a patient group that was less exposed to SARS-CoV-2 infection and did not experience the same complications as adults. Most infected infants at the time of diagnosis were asymptomatic or had a mild course, with only 10% experiencing severe symptoms. This situation may have supported a more stable approach to empirical antibiotic therapy in neonatal units. However, it should be noted that the amount of available data from these units was limited, which hinders a comprehensive assessment of the pandemic’s impact on microbial resistance [31,32].

Our analysis revealed borderline statistically significant mortality differences between *E. coli* cases and other pathogens (*p* = 0.0547; RR 1.55, 95% CI 0.54–4.41). The wide confidence interval reflects limited statistical power due to small sample size (39 confirmed early-onset sepsis cases, 13 deaths total), typical for single-center neonatal studies. Although the small sample size (*n* = 39) limits statistical power, the observed difference remains clinically notable and is consistent with the findings of Stoll et al., who similarly reported higher mortality in Gram-negative infections among preterm infants, albeit without reaching statistical significance [1]. Importantly, our study did not demonstrate an association between ampicillin resistance and increased mortality in sepsis caused by *E. coli* strains resistant to ampicillin, aligning with the findings of Dustinn et al. This suggests that ampicillin resistance does not directly lead to poorer clinical outcomes when alternative active agents, such as aminoglycosides and third-generation cephalosporins, are available [19].

Based on our analysis, we can conclude that the recommended empirical combination therapy with ampicillin and an aminoglycoside may be effective in treating neonatal bloodstream infections until the etiological agent is identified. However, the high rate of ampicillin resistance among strains causing early-onset sepsis, particularly *E. coli*, must be taken into account, as in situations requiring the rapid bactericidal effect characteristic of beta-lactams, this regimen may be less effective than a cephalosporin–aminoglycoside combination or meropenem monotherapy. In such cases, the aminoglycoside’s activity alone may be insufficient to achieve an adequate therapeutic response. Stoll et al. suggest considering the empirical use of a broad-spectrum antibiotic, such as a cephalosporin or a carbapenem, in preterm very-low-birth-weight (VLBW) infants when there has been prolonged intrapartum exposure to ampicillin or in cases of prolonged preterm rupture of membranes (PPROM) [1].

This study has several limitations. Its single-center design and small number of EOS cases limit generalizability. The association between *E. coli* infection and mortality was only borderline significant, likely due to limited statistical power. Adjustment was limited to gestational age, preventing evaluation of other confounders. Finally, the retrospective design introduces some selection bias despite strict inclusion criteria.

## 5. Conclusions

The obtained results highlight the need for continued analysis of the etiology, antimicrobial susceptibility, and resistance patterns of pathogens causing neonatal sepsis. Systematic microbiological surveillance supports the development of a local pathogen profile, which forms the basis for assessing the adequacy of empirical therapy in a given center and enables timely responses to the evolving epidemiological situation. These findings also indicate the necessity of further improving diagnostic methods that can accelerate the detection of sepsis, allow rapid identification of the etiological agent of bloodstream infections, and facilitate the recognition of antimicrobial resistance mechanisms. As a consequence, clinicians will be able to modify antibiotic therapy earlier, ensuring that treatment is more targeted and effective. Moreover, such analyses provide a foundation for enhancing screening strategies in both pregnant women and newborns, contributing to better identification of risk factors for early-onset infections.

## Figures and Tables

**Table 1:** Distribution of pathogens in early-onset sepsis among preterm and term infants.

Pathogen	Preterm [*n* = 31]		Term [*n* = 8]		Corrected *p*-Value *
	*n*	%	*n*	%	
Gram-positive	6	19.4	6	75.0	0.01
*Streptococcus agalactiae*	2	6.5	5	62.5	0.02
*Enterococcus* spp.	0	0.0	1	12.5	1.00
*Listeria monocytogenes*	2	6.5	0	0.0	1.00
CoNS	2	6.5	0	0.0	1.00
Gram-negative	25	80.7	2	25.0	0.01
*Enterobacterales*	23	74.2	2	25.0	0.03
*Escherichia coli*	18	58.0	2	25.0	1.00
*Klebsiella* spp.	2	6.5	0	0.0	1.00
*Citrobacter* spp.	1	3.2	0	0.0	1.00
*Morganella morganii*	1	3.2	0	0.0	1.00
*Pantoea agglomerans*	1	3.2	0	0.0	1.00
Other Gram-negative	2	6.5	0	0.0	1.00
*Pseudomonas aeruginosa*	1	3.2	0	0.0	1.00
*Haemophilus influenzae*	1	3.2	0	0.0	1.00

* Bonferroni-corrected Fisher’s exact test comparing pathogen distribution between preterm and term infants. Percentages calculated within gestational age groups. CoNS, coagulase-negative staphylococci.

**Table 2:** Antibiotic susceptibility of bacterial isolates.

Antibiotic	Ampicillin, *n* (%)	Piperacillin/Tazobactam, *n* (%)	Cefotaxime, *n* (%)	Ceftriaxone, *n* (%)	Meropenem, *n* (%)	Amikacin, *n* (%)	Gentamicin, *n* (%)	Vancomycin, *n* (%)
**Gram-positive**								
*Streptococcus agalactaie* (7)	7/7 (100) *	7/7 (100) *	7/7 (100) *	7/7 (100) *	7/7 (100) *	NR	NR	7/7 (100)
*Enterococcus* spp. (1)	1/1	1/1	NR	NR	NR	0/1	0/1	1/1
*Listeria monocytogenes* (2)	2/2	-	NR	NR	2/2	-	-	-
*CoNS* (2)	0/2	1/2	1/2	1/2	1/2	1/2	1/2	2/2
**Gram-negative**								
***Enterobacterales***								
*Escherichia coli* (20)	2/20 (10)	18/20 (90)	18/20 (90)	18/20 (90)	20/20 (100)	19/20 (95)	19/20 (95)	-
*Klebsiella* spp. (2)	0/2	2/2	2/2	2/2	2/2	2/2	2/2	-
*Citrobacter* spp. (1)	-	1/1	1/1	1/1	1/1	1/1	1/1	-
*Morganella morganii* (1)	-	1/1	1/1	1/1	1/1	1/1	1/1	-
*Pantoea agglomerans* (1)	-	1/1	1/1	1/1	1/1	1/1	1/1	-
**Other Gram-negative**								
*Pseudomonas aeruginosa* (1)	-	1/1	-	-	1/1	1/1	1/1	-
*Haemophilus influenzae* (1)	1/1	1/1	1/1	1/1	1/1	IE	IE	-

* Susceptibility inferred from penicillin for GBS per EUCAST guidelines. Results are presented as the number and percentage of susceptible isolates. Percentages were calculated and reported only for pathogen groups with a higher number of isolates; for groups with small sample sizes, results are presented as fractions (*n*/N) to avoid statistical misinterpretation, *n*—number of isolates susceptible to the antibiotic, N—total number of isolates. S—Susceptible, standard exposure; I—Susceptible, increased exposure; R—Resistant; NR—Natural Resistance; IE—Insufficient Evidence (the organism is not an appropriate target for treatment); (-)—Agent not recommended for therapy and susceptibility testing not advised. For statistical analysis, categories R, NR, IE, and (-) were considered non-susceptible.

**Table 3:** Antibiotic resistance profiles of pathogens.

Antibiotic	GBS (A) [*n* = 7]	*E. coli* (B) [*n* = 20]	Other (C) [*n* = 12]	*p*-Value (A vs. B)	*p*-Value (A vs. C)	*p*-Value (C vs. B)
Ampicillin	0/7 (0%)	18/20 (90%)	4/8 (50%)	<0.001	0.231	0.115
Piperacillin/Tazobactam	0/7 (0%)	2/20 (10%)	1/10 (10%)	1.000	1.000	1.000
Cefotaxime	0/7 (0%)	2/20 (10%)	1/8 (13%)	1.000	1.000	1.000
Cetriaxone	0/7 (0%)	2/20 (10%)	1/8 (13%)	1.000	1.000	1.000
Meropenem	0/7 (0%)	0/20 (0%)	1/11 (9%)	1.000	1.000	1.000
Amikacin	-	1/20 (5%)	2/9 (22%)	1.000	1.000	0.660
Gentamicin	-	1/20 (5%)	2/9 (22%)	1.000	1.000	0.660
Vancomicin	0/7 (0%)	*-*	0/3 (0%)	1.000	1.000	1.000

Data are presented as fractions (*n*/N) and percentages. (-): antimicrobial agent is unsuitable for treatment or susceptibility testing is not recommended by EUCAST. GBS susceptibility to ampicillin, cefotaxime, and meropenem was inferred from benzylpenicillin susceptibility. A—GBS, B—E. coli, C—Other (other less frequent pathogens)—Fisher’s exact test with Bonferroni correction comparing Groups A and B, A and C, C and B. A statistically significant difference was observed only for ampicillin resistance (*p* < 0.001). No significant temporal trends were observed across the study period (Kendall’s tau, all *p* > 0.05).

**Table 4:** Mortality distribution by pathogen type in early-onset sepsis among term and preterm infants.

	Preterm			Term			All			
All N = 39	GBS	*E. coli*	Other	GBS	*E. coli*	Other	GBS	*E. coli*	Other	Adjust RR for *E. coli* vs. Other (95%CI)
	*n* = 2	*n* = 18	*n* = 11	*n* = 5	*n* = 2	*n* = 1	*n* = 7	*n* = 20	*n* = 12	
All deaths N = 13	0	9	3	0	1	0	0	10	3	1.55 (95% CI 0.54–4.41)
*p*-value *	0.4062			0.3750			0.0547			

* Fisher’s exact test comparing mortality across pathogen categories. Adjusted relative risk (Poisson regression with robust standard errors, gestational age-adjusted). Wide confidence interval reflects limited events (13 deaths total). Adjusted relative risk of death for Escherichia coli compared with other pathogens, presented with a 95% confidence interval. *n*—number of isolates in individual age groups, N—total number of isolates.

**Table 5:** Impact of ampicillin resistance on mortality in neonatal *E. coli* sepsis, stratified by gestational age.

Gestational Age Group	Resistant (*n*)	Resistant Deaths (*n*)	Susceptible (*n*)	Susceptible Deaths (*n*)	*p*-Value *	Adjusted RR (95%CI) (res vs. sus)	Adjusted RR *p*-Value
All	17	8	2	1	1.0000	1.01 (0.38–2.68)	0.990
Term	2	1	0	0	1.0000		
Preterm	15	7	2	1	1.0000	1.01 (0.53–1.91)	0.978

* Fisher’s exact test comparing mortality between resistant and susceptible groups. Adjusted RR (95% CI): Adjusted relative risk of death for resistant versus susceptible strains, with 95% confidence interval, estimated using Poisson regression. Resistant (*n*): Number of neonates infected with E. coli strains resistant to ampicillin. Resistant Deaths (*n*): Number of deaths among neonates infected with resistant strains. Susceptible (*n*): Number of neonates infected with E. coli strains susceptible to ampicillin. Susceptible Deaths (*n*): Number of deaths among neonates infected with susceptible strains. Res—resistant, sus—susceptibility.

## Data Availability

The data presented in this study are available on request from the corresponding author.
